# Regional differences in socioeconomic trends: The spatiotemporal evolution from individual cities to a megacity region over a long time series

**DOI:** 10.1371/journal.pone.0244084

**Published:** 2020-12-21

**Authors:** Zhiming Sun, Xianglong Chen, Hanfa Xing, Hongtao Ma, Yuan Meng

**Affiliations:** 1 School of Geography, Yunnan Normal University, Kunming, Yunnan, China; 2 Center for Myanmar Studies of Yunnan Normal University, Kunming, Yunnan, China; 3 School of Geography, South China Normal University, Guangzhou, Guangdong, China; 4 SCNU Qingyuan Institute of Science and Technology Innovation Co., Ltd., Qingyuan, Guangdong, China; 5 Guangdong Normal University Weizhi Information Technology Co., Ltd., Qingyuan, Guangdong, China; 6 Department of Land Surveying and Geo-Informatics, The Hong Kong Polytechnic University, Kowloon, Hong Kong; Institute for Advanced Sustainability Studies, GERMANY

## Abstract

Regional differences in socioeconomic factors are important for assessing the regional development of an area. While much research has focused on the overall patterns of regional differences within independent cities and areas, the hierarchical spatiotemporal structures of megacity regions have seldom been discussed. To fill this gap, this paper investigates the multilevel regional differences within megacity regions. Employing GDP, population and total retail sales as socioeconomic indicators, the spatiotemporal patterns of socioeconomic trends are identified. A hierarchical clustering approach that utilizes socioeconomic similarities is proposed for the identification of the spatiotemporal patterns of individual cities. At the megacity regional level, gravity centers and pathways are constructed to evaluate spatial imbalances and temporal change intensities. Taking the Guangdong-Hong Kong-Macao Greater Bay Area (GBA) as its study area, this research produces results that show diverse spatiotemporal patterns among the individual cities, revealing high/low starting point and high/low growth rate modes in terms of city interactions. From the perspective of the entire GBA, the spatial imbalance of GDP is the highest of the factors, followed by the spatial imbalance of the total retail sales of the region and, finally, by that of its population. Total retail sales exhibit the highest level of temporal change intensity, followed by GDP and population. In terms of the contribution of the various cities to the overall regional changes, Guangzhou, Shenzhen and Hong Kong dominate the spatiotemporal changes in the gravity centers, while Foshan and Dongguan show significant potential to contribute to these socioeconomic patterns. These results provide effective guidance for the sustainable development of megacity regions.

## Introduction

Regional differences are often linked to divisions in socioeconomic development [1, 2]. Because of the “reform and opening-up” policy, China has been experiencing rapid changes in recent decades, and the markets related to productivity vary among cities and further exhibit different levels of economic progress [3]. The existing research has investigated many types of regional differences including residential environments [4, 5], housing prices [6], environmental pollution and transportation [7, 8]. These spatiotemporal socioeconomic differences have been regarded as one of the significant challenges in China [9], especially in megacity regions with urban agglomerations, such as the Guangdong-Hong Kong-Macao Greater Bay Area (GBA). The dynamic processes of public resource allocation and transportation organization have created various patterns of socioeconomic trends among megacity regions [10], and these trends have further promoted more regional differences among the cities that interact with one another in megacity regions. Identifying the spatiotemporal socioeconomic differences within megacity regions in China is required to obtain a better understanding of how megacity regions form in terms of regional economic differences [11].

As the megacity region with the most rapid economic and innovation-related development in China, the GBA shows significant variations in its spatiotemporal socioeconomic trends. Guided by the Outline Development Plan for the Guangdong-Hong Kong-Macao Greater Bay Area proposed by the Chinese government, the innovation-driven connections and cooperation in terms of the socioeconomics among the cities in the GBA are much deeper, leading to rapid urbanization processes, especially in underdeveloped areas. These varying socioeconomic distributions and connections among cities make it necessary to explore the spatiotemporal regional differences in the GBA. The existing research has focused the regional differences in the GBA in terms of various aspects. Zhou et al. found that the differences among regional geographic locations can lead to different development patterns within the cities of these regions [12]; this phenomenon causes the imbalanced economic development observed in the GBA. Wen found that there are large differences between the east and west sides of the Pearl River Estuary in the GBA in terms of economic strength, stage of industrial development, and number of permanent residents [13]. In addition, Ni et al. found that the socioeconomic distribution of the GBA exhibits an imbalanced pattern between its east and west sides, as well as a parallel trend between its north and south [14]. The eastern and western regions of the GBA show large and changing gradient differences in terms of economic scale, energy level, and industrial structure.

Due to the regional differences occurring in the GBA, effective measurements are required to quantify its socioeconomic variations. In particular, gross domestic product (GDP) and total retail sales provide evidence to capture the pulse of the economy and the corresponding expansion or contraction conditions by measuring the total monetary market values and individual consumption abilities [15], while the population growth will contribute to economic growth and reduce income inequalities [16]. In fact, GDP, total retail sales and population have been utilized to measure regional differences in many studies. For instance, Lozano et al. utilized GDP and population to examine the uneven distribution of primary energy consumption and greenhouse gas emissions [17]. Krausmann et al. analyzed the relation between the overall size and composition of global material flows and global economic and population changes [18]. The previous research has also evaluated the effect of natural environmental factors on populations, revealing a high correlation between natural environments and population distribution [19]. In addition, Bakos focused on the retail commerce occurring in the digital market and compared multiple aspects of this market with aspects of the traditional market [20]. Regarding spatiotemporal changes in total retail sales, Liu explored various types of retail sales occurring between 1954 and 1963 in large metropolitan regions using a retail sales model [21]. Despite the above measurements of GDP, population and total retail sales, the hierarchical structures of regional economic differences and their pathways from the entire megacity region to individual cities have not been comprehensively understood. In fact, the research has indicated that due to the development of communication technology and transportation networks, the regional hierarchical structure has become the most refined and appropriate representation of the urban system structure and regional heterogeneity [22].

To identify the hierarchical structures of megacity-level regional differences, effective approaches for measuring the spatiotemporal interactions among cities and megacity regions are required. While clustering methods can be effectively used to identify the similarities among certain economic characteristics to evaluate the interactions among cities [23, 24], gravity models focus on depicting the moving pathways, including distance and directions, of the centers of specific socioeconomic characteristics [25]. From the perspective of the entire megacity region, the movement of the gravity center represents increasing trends of the socioeconomic indicators within certain distances and directions in the GBA, indicating the spatiotemporal differences driven by the changes in GDP, total retail sales and population. For individual cities, the contributions to the movement of the gravity centers of independent cities can be used to further explore their interactions. Previous studies have utilized the gravity model to analyze the migration trajectory of the gravity center of China's rural industrial distribution [26, 27]. Moreover, the spatial distribution patterns and dynamic change processes of regional development indexes, such as the population gravity center [28], GDP gravity center [29], pollution gravity center [30], land use gravity center [11], tourism gravity center [9] and grain production gravity center [31], and the integration or interactions of multiple factors [32, 33], have been analyzed in the literature. These approaches show the potential to be effectively applied to analyze the multilevel regional economic differences in megacity regions. However, the gravity model has seldom been applied in investigating the hierarchical spatiotemporal structures of mega-urban regions in long-term series.

Based on the above discussion, challenges still exist in understanding how socioeconomics contribute to the regional differences within the GBA megacity region. First, it is necessary to explore the effective measurements of GDP, total retail sales and population among the interactions of individual cities. Second, how to depict the spatiotemporal differences and their movement pathways in terms of megacity region-level socioeconomics needs to be investigated. Third, depicting the hierarchical structures of regional differences, from individual cities to the entire GBA megacity region, remains a question. To fill these gaps, this study proposes a new framework to investigate the spatiotemporal evolution of the regional economic differences within the megacity regions of the GBA. In particular, among individual cities, the socioeconomic trends and temporal similarities of spatiotemporal interactions are measured based on GDP, population and total retail sales; for the entire GBA megacity region, the movement pathways of the gravity centers of the socioeconomic indicators are measured in terms of distances and directions; from individual cities to the megacity region, the contributions of major cities are analyzed. The proposed framework and findings can provide guidance to help the government develop sustainable megacity regions.

## Study area and data sources

### Study area: Guangdong-Hong Kong-Macao Greater Bay Area

**Fig 1:**
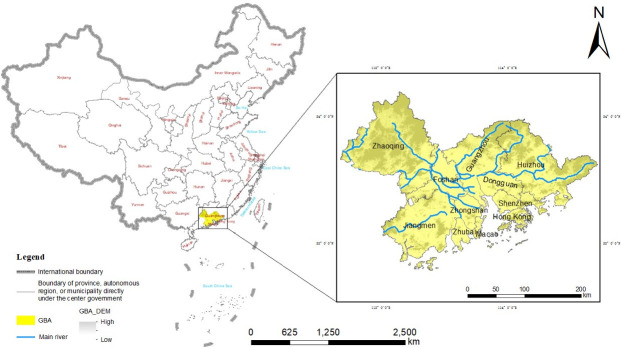
Location of the GBA in China. Reprinted background map from the National Catalogue Service for Geographic Information (www.webmap.cn) under a CC BY license, with permission from the National Geomatics Center of China, original copyright 2020.

The GBA megacity region, located in southern China, is selected as the study area. The GBA megacity region consists of 9 cities, namely, Guangzhou, Foshan, Zhaoqing, Shenzhen, Dongguan, Zhuhai, Jiangmen, Zhongshan and Huizhou, as well as 2 special administrative regions, namely, Hong Kong and Macao (shown in Fig 1). The GBA encompasses the main area of the Pearl River Delta and is considered one of the most important components of the world-class urban agglomeration in China and in enabling the country’s participation in global competition. With a total area of 56,000 square kilometers, the GBA is the fourth-largest megacity region in the world, after the New York Bay Area and the San Francisco Bay Area in the United States and the Tokyo Bay Area in Japan [34]. In 2018, the total population of the GBA reached 70 million; additionally, its GDP exceeded 10 trillion yuan, accounting for 12.17% of the national economy. The total GDP of this region ranks 11th in the world.

### Data sources from 2000 to 2018

Statistical data corresponding to nineteen periods from 2000 to 2018 are selected for analysis. The total population index is used to represent the number of people in the area, the regional GDP indicator is used to represent economic development, and the total retail sales of social consumer goods is used to represent the consumption level. These statistics are taken from the annual data analysis of China Economic and Social Big Data Research Platform (http://data.cnki.net/YearData/Analysis), the National Bureau of Statistics of China (http://www.stats.gov.cn/tjsj/), the Statistics Department of the Government of the Hong Kong Special Administrative Region (https://www.censtatd.gov.hk/), the Statistics and Census Bureau of the Government of the Macao Special Administrative Region (https://www.dsec.gov.mo/zh-MO/), and the statistical yearbooks and bulletins of each administrative region within the GBA for the relevant years. The monetary units of the Hong Kong and Macao economic statistics have been converted into RMB units according to the average exchange rate data at the end of each quarter of each year, in which the exchange rate data come from the China Foreign Exchange Trading System-National Interbank Funding System (http://www.chinamoney.com.cn/), and the State Administration of Foreign Exchange (http://www.safe.gov.cn/). It should be noted that as some changes have taken place in the administrative districts in the GBA since 2000, some statistical data are missing in these changing districts. Thus, biases could exist in calculating city-level or even smaller-scale socioeconomic indicators during the period of 2000–2018.

**Fig 2:**
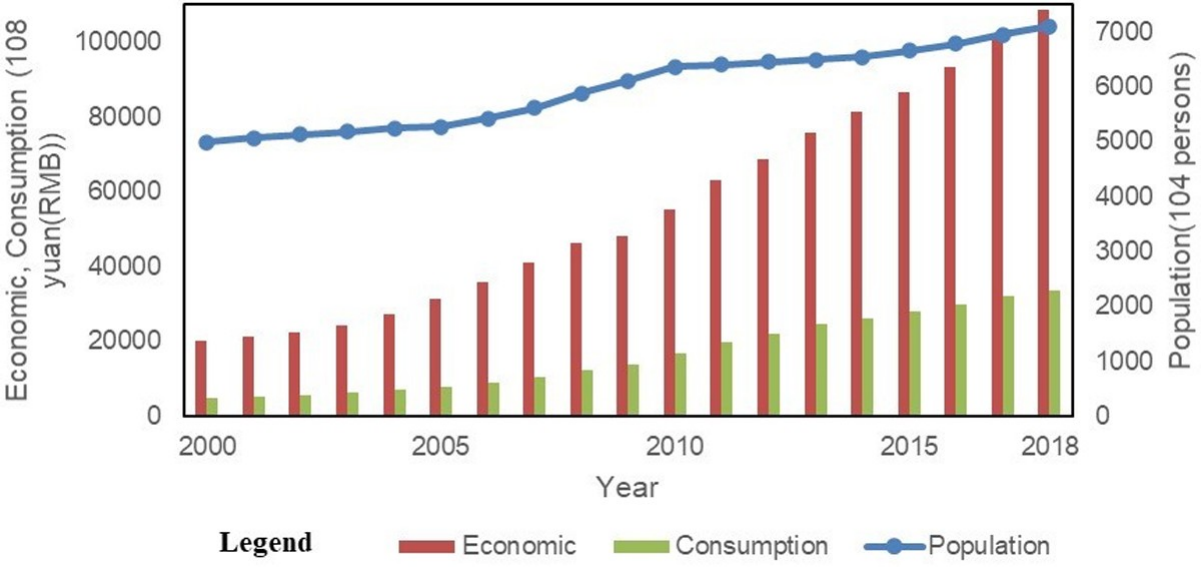
The development of the population, GDP and total retail sales of the GBA from 2000 to 2018.

As shown in Fig 2, the socioeconomic indicators of the GBA changed dramatically from 2000 to 2018. The population of the GBA increased from 49.99 million in 2000 to 71.09 million in 2018. During the 18 year period, the total population of the area increased by 21.1 million, with an average annual increase of 1.17 million. In 2018, the GDP of the GBA reached 10,865.84 billion yuan (5.38 times that of 2000) and exhibited an average annual growth rate of 24.31%. The total retail sales of social consumer goods in the GBA reached 3,364.25 billion yuan in 2018 (7.88 times that of 2000) and exhibited an average annual growth rate of 38.2%; this growth rate was higher than that of the regional GDP for that year.

## Methods

**Fig 3:**
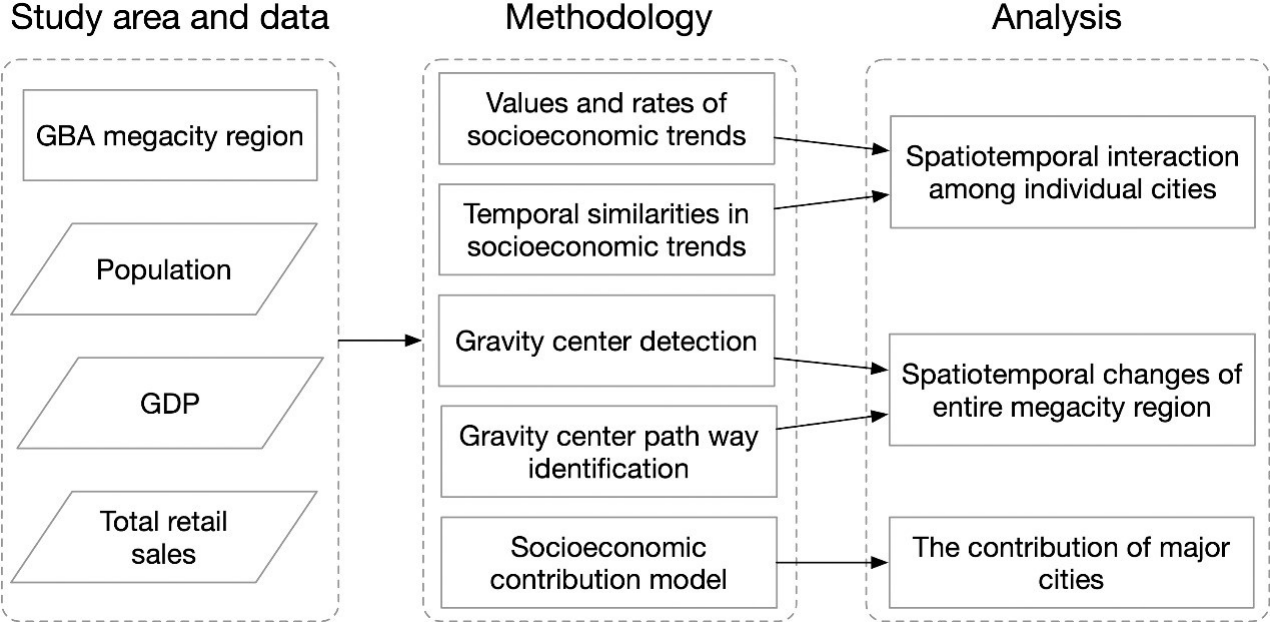
The overview of the research framework.

The overall framework used for this study is shown in Fig 3. Taking the GBA megacity as its study area, this paper adopts three socioeconomic factors, namely, population, GDP and total retail sales, to investigate the spatiotemporal patterns of the socioeconomic trends occurring from 2000 to 2018 on different levels. Through quantifying the values, rates and temporal similarities of the socioeconomic trends in the area, the spatiotemporal interactions among the individual cities are analyzed. To examine the socioeconomic trends of the entire megacity region, gravity center detection and gravity center pathway identification are proposed. Moreover, the socioeconomic contribution model is applied to identify the contributions of the major cities to the area’s overall socioeconomic trends.

### Spatiotemporal interactions among the individual cities

The regional differences in terms of the three socioeconomic indicators, namely, GDP, total retail sales and population, are involved in the exploration of the spatiotemporal interactions between the individual cities. Both the growth values and the growth rates of these three indicators are considered when detecting the changes in various trends; subsequently, the similarities among the indicators of the different cities are quantified using a hierarchical clustering method.

To detect the changes in the trends of the socioeconomic indicators, this paper presents their growth values and growth rates with a linear graph and a cumulative percentage histogram; in the graph and the histogram, the growth values of the indicators are depicted annually, and the growth rates correspond to four periods, namely, 2000–2005, 2005–2010, 2010–2015 and 2015–2018. To quantify the similarities among the cities, the hierarchical clustering method is adopted in this paper. First, the indicators are subjected to z standardization processing to eliminate the influence of dimensional and order-of-magnitude differences among them on the calculation results. Then, the temporal similarities among the socioeconomic indicators are defined as the distances between the clusters obtained through the hierarchical clustering method; these distances are calculated using the square European distance as follows:

$$d_{ij}=\sum_{k=1}^{n}{\left(x_{ki}-x_{kj}\right)}^{2}$$ (1)

Where *d*_*ij*_ represents the similarity coefficient between city *i* and city *j*, *x*_*ki*_ represents the standardized value of a certain socioeconomic index in city *i*, *x*_*kj*_ represents the standardized value of a certain socioeconomic index in city *j*, and _*n*_represents the total number of socioeconomic indexes. Then, the *n* × *n* Euclidean distance matrix is constructed as follows:

$$D=\left(\begin{array}{ccc} d_{11} & \ldots & d_{1n}\\ \vdots & \ddots & \vdots \\ d_{n1} & \cdots & d_{nn} \end{array}\right)$$ (2)

Where *D* is a symmetric matrix, and *n* cities can be classified according to *D*. On this basis, a hierarchical binary tree is proposed using the hierarchical clustering method. Specifically, each city is treated as an individual cluster; as the variance (or measured dissimilarity) increases in the upper portions of the hierarchy, pairs of clusters merge to create new clusters. Thus, cities with similar development characteristics are connected to one another in the tree.

### Spatiotemporal changes in the entire megacity region

The spatiotemporal changes within the entire megacity region are investigated by analyzing the gravity centers of the socioeconomic indicators and identifying their gravity center paths. In particular, the gravity centers of the socioeconomic indicators are detected by delineating the locations of the gravity centers and the characteristics of the spatial distributions, while the gravity center pathways are identified by determining their directions and lengths.

#### Gravity center detection

*Spatial distribution of the gravity centers*. The term gravity center refers to the various points in an object generated by the gravity [35]; this factor is calculated as the average value of the geometric coordinates of all the cities in a region [36]. In this way, the socioeconomic indicators of the area are employed to derive the gravity centers of GDP, total retail sales and population. This modified method quantifies the value of each of these attributes within each city and uses these values as weights to calculate the weighted regional average gravity center of the three indicators as follows [34]:

$$X=\frac{\sum_{i=1}^{n}P_{i}x_{i}}{\sum_{i=1}^{n}P_{i}},Y=\frac{\sum_{i=1}^{n}P_{i}y_{i}}{\sum_{i=1}^{n}P_{i}}$$ (3)

where *n* is the total number of cities in the study area, and *x*_*i*_ and *y*_*i*_ are the longitude and latitude coordinates, respectively, of the *i*-th city. *p*_*i*_ is the quantified value of the *i*-th city, and *X* and *Y* are the coordinates of the gravity centers corresponding to GDP, total retail sales and population.

*Spatial distribution of the standard deviation ellipses*. Based on the calculated gravity centers, standard deviation ellipses (SDEs) are applied to reveal the spatial distribution characteristics of the gravity centers in terms of the spreading range, dispersion, direction and shape; this calculation is as follows [37]:

$$\bar{X_{w}}=\frac{\sum_{i=1}^{n}w_{i}x_{i}}{\sum_{i=1}^{n}w_{i}},\bar{Y_{w}}=\frac{\sum_{i=1}^{n}w_{i}y_{i}}{\sum_{i=1}^{n}w_{i}}$$ (4)

$$\sigma_{X}=\sqrt{\frac{\sum_{i=1}^{n}{\left(w_{i}\bar{x_{i}}\cos\theta -w_{i}\bar{y_{i}}\sin\theta \right)}^{2}}{\sum_{i=1}^{n}w_{i}^{2}}},\sigma_{y}=\sqrt{\frac{\sum_{i=1}^{n}{\left(w_{i}\bar{x_{i}}\sin\theta -w_{i}\bar{y_{i}}\cos\theta \right)}^{2}}{\sum_{i=1}^{n}w_{i}^{2}}}$$ (5)

$$\tan\theta =\frac{(\sum_{i=1}^{n}w_{i}^{2}\bar{x_{i}^{2}}-\sum_{i=1}^{n}w_{i}^{2}\bar{y_{i}^{2}})+\sqrt{{\left(\sum_{i=1}^{n}w_{i}^{2}\bar{x_{i}^{2}}-\sum_{i=1}^{n}w_{i}^{2}\bar{y_{i}^{2}}\right)}^{2}+4\sum_{i=1}^{n}w_{i}^{2}\bar{x_{i}^{2}}\bar{y_{i}^{2}}}}{2\sum_{i=1}^{n}w_{i}^{2}\bar{x_{i}^{2}}\bar{y_{i}^{2}}}$$ (6)

where $\bar{X_{w}}$ and $\bar{Y_{w}}$ represent the center of a spatial distribution, $\bar{x_{i}}$ and $\bar{y_{i}}$ represent the relative coordinates of the *i*-th coordinate point from the gravity center of the spatial distribution, and *n* is the total number of cities. The weight is represented by *w*_*i*_. This paper takes the population, GDP and total retail sales of each city as its weights. The azimuth angle of the SDE is represented by *θ*; this indicates the angle formed by the long axis of the SDE when it is rotated clockwise from the positive north direction. The lengths of the *x* and *y* axes of the SDE are represented by *σ*_*x*_ and *σ*_*y*_.

#### Gravity center pathway analysis

*Direction of gravity center movement*. The population, GDP and total retail sales in the region are constantly developing and changing. If the development of a certain factor in the region is not balanced, the regional gravity center of that factor will move in the direction of "high density"; its direction angle is then calculated as follows [38]:

$$\theta_{(k+1)-k}=\frac{n\pi }{2}+\arctan\theta (\frac{y_{k+1}-y_{k}}{x_{k+1}-x_{k}})$$ (7)

where the correction coefficient *n* is (0, 1, 2); *x*_*k*+1_−*x*_*k*_ and *y*_*k*+1_−*y*_*k*_ represent the changes in the longitude and the latitude of the barycenter coordinate between the *k*−-th year and the *k+*1 year, respectively; and *θ*_(*k*+1)−*k*_ represents the change in the barycenter movement angle (−180° ≤ *θ* ≤ 180°) between the *k*-th year and the *k+*1 year. When *θ* = 0°, the gravity center moves due east. When *θ* = 180°, the gravity center moves in the positive west direction. When 0° < *θ* < 90°, the gravity center moves toward the northeast. When 90° < *θ* < 180°, −180° < *θ* < −90° and −90° < *θ* < 0°, the gravity center moves toward the northwest, southwest and southeast, respectively.

*Distance of gravity center movement*. The distance of the gravity centers’ movement represents the degree of the changes that occurred in the regional factors of each city during a certain time period; this is jointly determined by these factors’ development within all the cities of the research area. The calculation of this distance is as follows [29]:

$$d_{(k+1)-k}=C\times \sqrt{{\left(X_{k+1}-X_{k}\right)}^{2}+{\left(Y_{k+1}-Y_{k}\right)}^{2}}$$ (8)

Where *C* is a constant equal to 111 km that represents the coefficient of conversion from the coordinate unit of the earth's surface (degree) to the plane distance (km); *X*_*k*+1_−*X*_*k*_ and *Y*_*k*+1_−*Y*_*k*_ and represent the changes in the longitude and the latitude of the gravity center coordinates between year *k* and year *k+*1, respectively; and *d*_(*k*+1)−*k*_ represents the distance that a gravity center moved between year *k* and year *k+*1.

### Contributions of major cities

To explore the influence of different cities on the overall regional differences, this paper compares the net growth of the three indicators within the different cities of the GBA in 2018 with the net growth of the same indicators in 2000; additionally, it analyzes the contribution of each city during different periods. The socioeconomic contribution model enables the direct separation of a specific socioeconomic index from the overall economic growth of an area [39]. It is utilized to separately identify the contribution of each city and evaluate this contribution in terms of the overall development of a megacity region. The calculation is as follows:

$$g_{i}^{t}=\frac{G_{i}^{t}}{\sum_{i=1}^{n}G_{i}^{t}}\times 100\%(G_{i}^{t}=Y_{1}^{t}-Y_{0}^{t})$$ (9)

where $Y_{0}^{t}$ and $Y_{1}^{t}$ represent a certain socioeconomic index value for the base period and current period, respectively; $G_{i}^{t}$represents the growth of the specified socioeconomic index; *i* represents the city examined; and *t* represents the index type. The direct influence of city *i* on the specified index of the megacity region is represented by $g_{i}^{t}$; thus, this factor indicates the degree of that city’s contribution.

## Results

### Spatiotemporal evolution among the individual cities

#### Changes in the population, GDP and total retail sales trends of the individual cities

**Fig 4:**
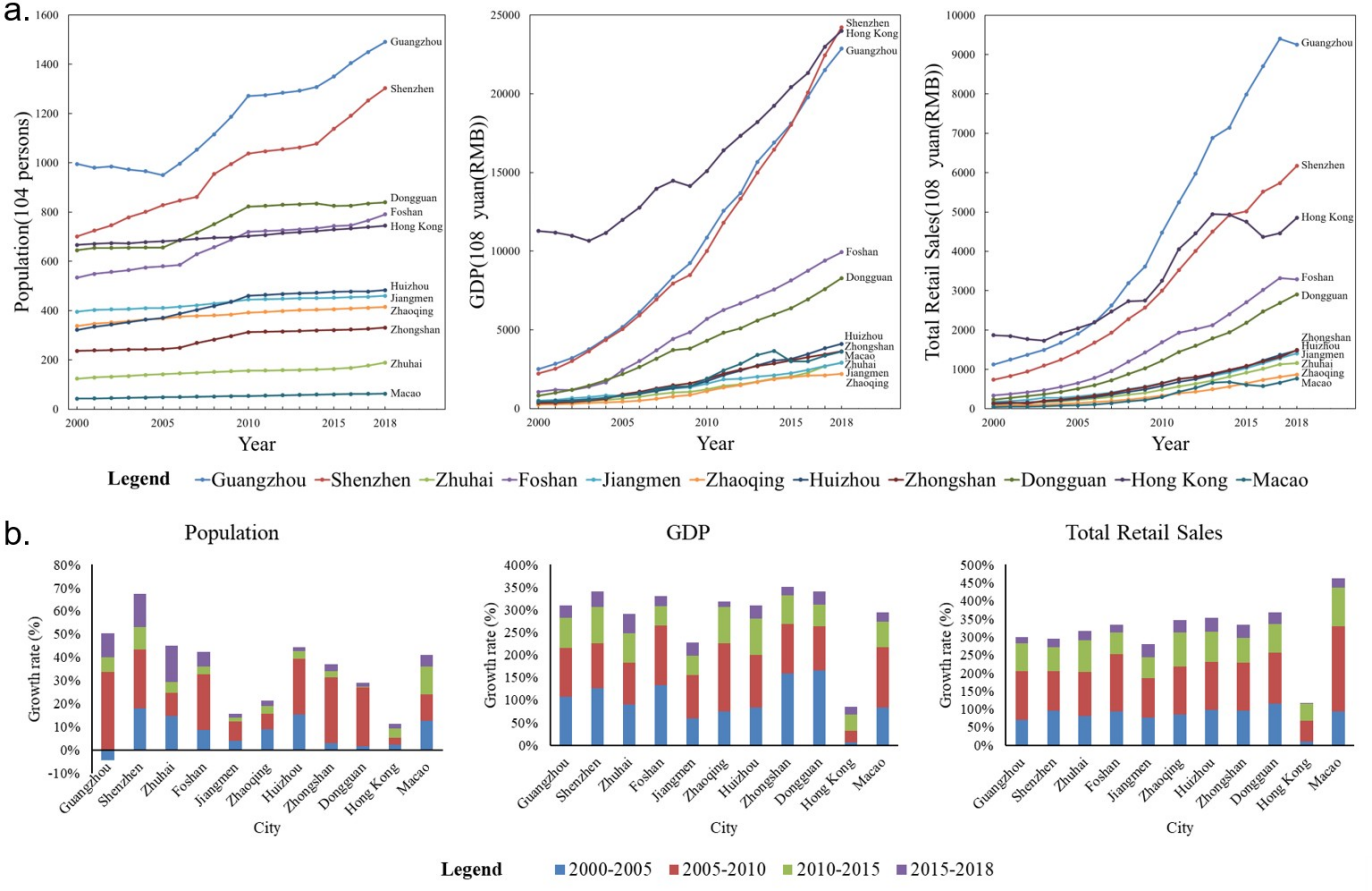
The changes in (a) and growth rates of (b) the populations, GDP and total retail sales of the different cities.

As shown in Fig 4(A), Guangzhou and Shenzhen had the largest populations from 2000 to 2018; Dongguan, Hong Kong and Foshan followed these areas in terms of population, and the other cities including Huizhou, Jiangmen, Zhaoqing, Zhongshan, Zhuhai and Macao, had relatively small populations. In terms of economics and consumption levels, the different cities show similar growth characteristics. Guangzhou, Shenzhen and Hong Kong exhibit the highest levels of economic and consumption development. Foshan and Dongguan are second in terms of this development, while the remaining cities including Huizhou, Zhongshan, Macau, Zhuhai, Jiangmen and Zhaoqing, rank third. Due to the differences in the population carrying capacities and economic carrying capacities among the different regions, the absolute value gaps between the social development indicators of the different regions within the GBA widened during the past 18 year period.

The changing trends of the growth rates can be clearly observed in Fig 4(B). From 2000 to 2018, the cities in the GBA achieved positive population growth. Among them, Shenzhen has the highest population growth rate, proving its strong demographic appeal. The population growth rates of Jiangmen, Zhaoqing, and Hong Kong are much lower. Obviously, these three cities are not preferred places for the migrant population. Except for Hong Kong, the overall differences in the growth rates of GDP and total consumption of each city from their original basis are not obvious. The reason that Hong Kong exhibits a lower development speed is that its original economic and consumption levels are higher; thus, it has less room to increase than other cities do, which explains the slower rate of Hong Kong's development and the rapid advancement of the other cities in the area. However, Hong Kong still plays a strong leading role among these cities.

#### Similarity of spatiotemporal evolution

**Fig 5:**
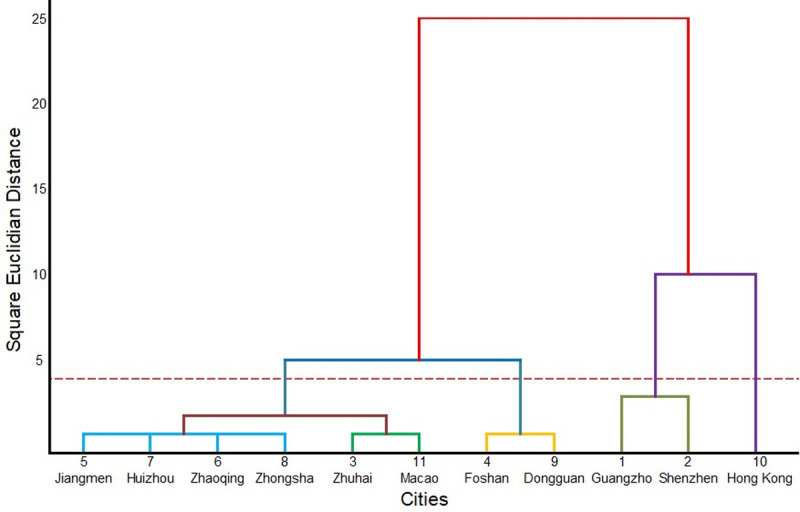
Tree diagram of the hierarchical clustering analysis.

The results of the hierarchical clustering method are shown in Table 1 and Fig 5. Specifically, the entire GBA megacity region can be divided into two categories as follows: Guangzhou, Shenzhen, and Hong Kong can be placed in a category corresponding to a high level of development, and the other nine cities can be categorized as relatively underdeveloped in terms of population, GDP and total retail sales. According to the different modes of urban socioeconomic development, when the square Euclidean distance is 4, these cities can be further divided into four non-ranking categories, as follows: Hong Kong is placed in its own category, as it has a high starting point and a low growth rate; Guangzhou and Shenzhen are placed in the second category, which is characterized by high starting points and high growth rates; Dongguan and Foshan are placed the third category, which is characterized by low starting points and high growth rates; and the remaining six cities belong to the fourth category, which is characterized by low starting points and low growth rates. The fourth category can be further subdivided into categories containing 1) Macao and Zhuhai and 2) Zhongshan, Zhaoqing, Huizhou, and Jiangmen.

**Table 1:** Hierarchical clustering results.

Stage	Cluster Combined		Coefficients	Stage Cluster First Appears		Next Stage
	Cluster 1	Cluster 2		Cluster 1	Cluster 2	
1	5	7	0.500	0	0	2
2	5	6	1.301	1	0	5
3	3	11	2.322	0	0	6
4	4	9	3.142	0	0	8
5	5	8	3.317	2	0	6
6	3	5	14.305	3	5	8
7	1	2	24.583	0	0	9
8	3	4	45.202	6	4	10
9	1	10	86.162	7	0	10
10	1	3	224.905	9	8	0

**Fig 6:**
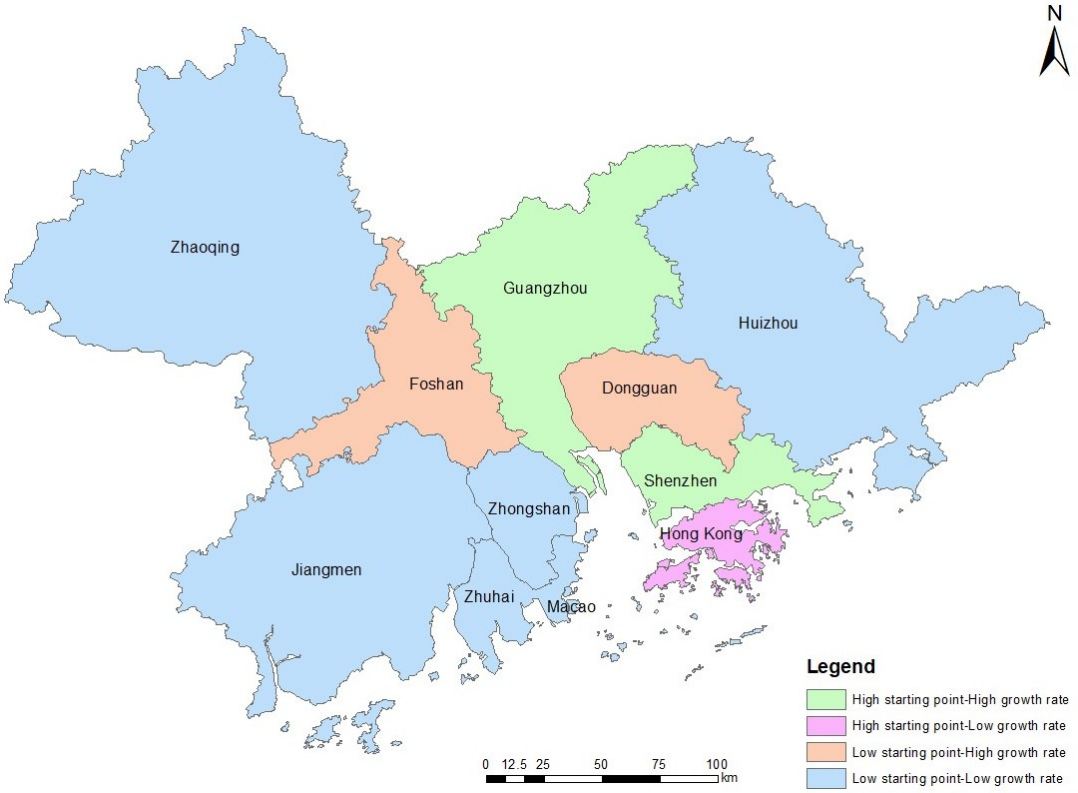
Classification of the urban development characteristics of the cities in the GBA. Reprinted background map from the National Catalogue Service for Geographic Information (www.webmap.cn) under a CC BY license, with permission from the National Geomatics Center of China, original copyright 2020.

The classification of the individual cities based on the above spatiotemporal similarity patterns is shown in Fig 6. Cities with similar patterns in terms of population, GDP, and total retail sales are classified into one category. Guangzhou, Shenzhen, and Hong Kong, which are the three developed cities, are naturally separated from the other cities by virtue of their large populations and GDP volumes. Because the development speed of Hong Kong's three indicators is obviously different from that of the indicators of Guangzhou and Shenzhen, Hong Kong will be separated from these two cities when the cities are further subdivided. Dongguan and Foshan are the second-class cities in the GBA, and their development characteristics are also different from those of the remaining cities. Overall, the clustering results effectively classify each individual city and show that some of the cities in the GBA exhibit the same development characteristics as others.

### Spatiotemporal evolution of the entire megacity region

#### Spatial distribution patterns of the gravity centers

**Fig 7:**
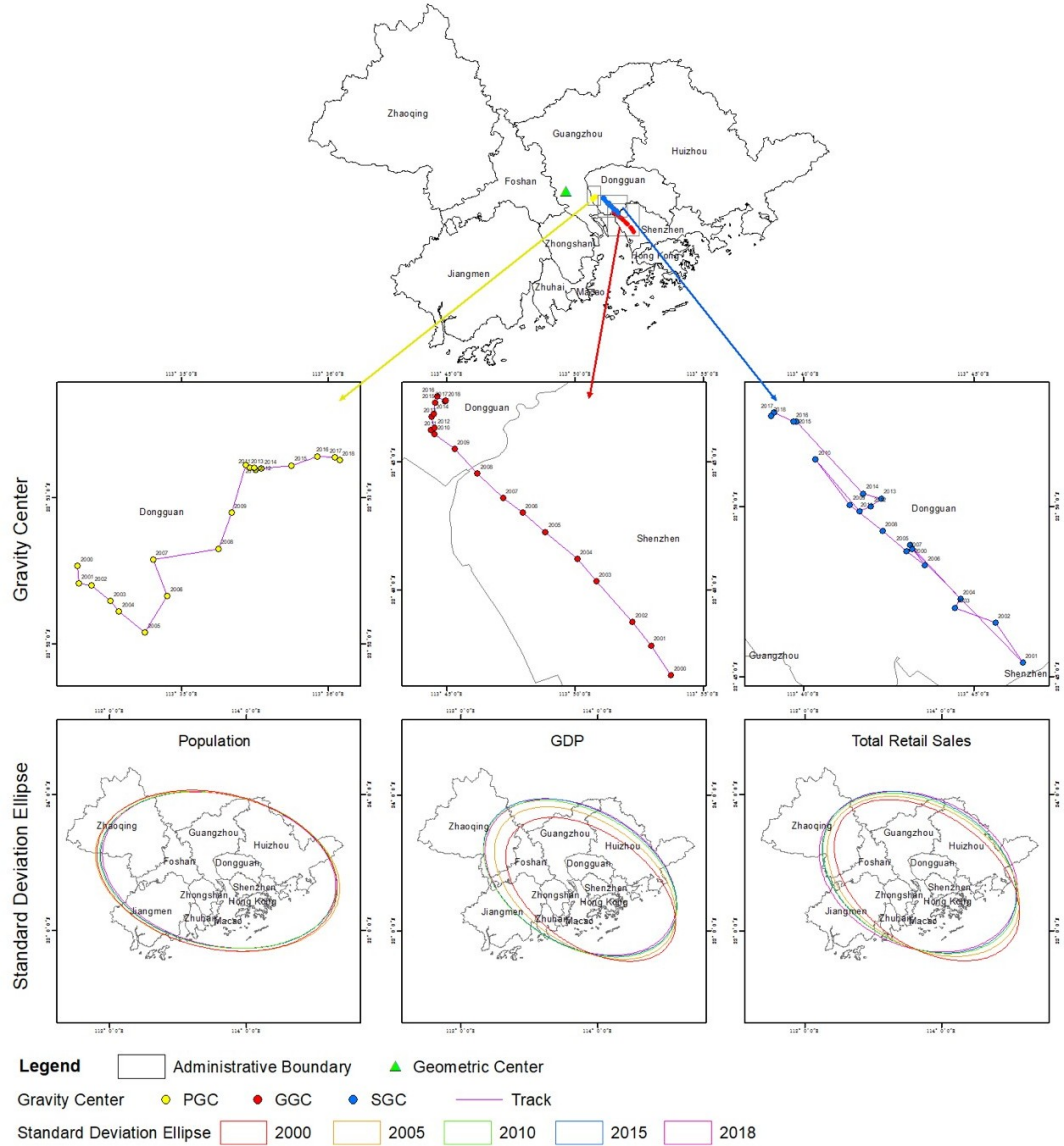
Gravity centers and SDEs of the population, GDP and total retail sales of the GBA. Reprinted background map from the National Catalogue Service for Geographic Information (www.webmap.cn) under a CC BY license, with permission from the National Geomatics Center of China, original copyright 2020.

As shown in Fig 7, compared with the geographical coordinates of the geometric center of the GBA in the Panyu District of Guangzhou, Guangdong, the population gravity centers of the GBA for the time period from 2000 to 2018 is located in Shatian Town of Dongguan; the GDP gravity centers for the same time period is located in the Bao'an District of Shenzhen; and the gravity centers of total retail sales for this time period is located in Humen Town of Dongguan. Assuming that the average gravity centers are independent points, starting from the geometric center of the area and traveling southeast, one would pass through the average gravity center of the population, then the average center of total retail sales, and finally the average center of GDP. From 2000 to 2018, the population gravity center, GDP gravity center, and total retail sales gravity center of the GBA did not coincide with the regional geometric center of the area. These three gravity centers remained southeast of the regional geometric center during that time period and are different average distances from the geometric center. This shows that the regional population, GDP and total retail sales of the GBA are unevenly distributed. Among these factors, the GDP of the area shows the highest degree of imbalance, followed by total retail sales and population. The three gravity centers are all located east of the regional geometric center, indicating that the eastern cities of the GBA have higher population densities, GDP and total retail sales than the western cities.

The SDEs further illustrate the degree of imbalances among the three indicators. Specifically, the distributions generally trend in the direction of "southeast-northwest"; additionally, their azimuth angles mainly range between 102 and 125 degrees, and they are all greater than 90 and less than 135. This shows that the population, GDP and total retail sales in the east-west direction are slightly higher than those in the north-south direction; this trend is also related to the shape of the GBA. During the 18 year period studied, the SDE of the population pattern in the GBA remained relatively the same, while the SDE of the GDP pattern and the total retail sales pattern clearly moved toward the northwest. From the evolutionary trend in terms of the long and short axes of the SDE, it can be observed that the lengths of the long and short axes of the population pattern SDE are decreasing; that is, this SDE are shrinking toward the central city. However, the lengths of the long and short axes of the GDP pattern SDE show an increasing trend that is the opposite of the population pattern, indicating that the economy of the GBA expanded from 2000 to 2018 but that the population was more concentrated during that time.

#### Evolutionary process of the three gravity centers

From 2000 to 2018, the geographic coordinates of the population gravity center ranged from 113°34′17″ to 113°36′4″E and from 22°52′4″ to 22°53′15″N, all located in Dongguan. Compared with its location in 2000, the population gravity center had moved 3.34 kilometers to the northeast by 2018. It further deviated from the geometric center of the region in terms of longitude and latitude. In general, the evolution of the population gravity center of the GBA from 2000 to 2018 can be divided into a large-scale phase of movement from 2000 to 2010 and a small-scale phase of movement from 2010 to 2018. During the former stage, the gravity center of the population moved to the southeast and then to the northeast, exhibiting a “V” shape. During the latter stage, the gravity center of the population moved eastward, and the magnitude of this movement was significantly smaller than that of the former stage. During this period, the maximum range of movement exhibited by the population gravity center was 2.42 kilometers northeast from 2005 to 2010.

During the 18 years studied, the geographical coordinates of the GDP gravity center ranged from 113°44′21″ to 113°53′45″E and from 22°36′38″ to 22°47′34″N and were located in Dongguan and Shenzhen. Compared with its location in 2000, the GDP gravity center had moved 24.95 kilometers to the northwest by 2018. By the end of the study period, it was far from the regional geometric center in terms of latitude and longitude. In general, the evolution of the GDP gravity center of the GBA from 2000 to 2018 can be divided into a large-scale phase of movement from 2000 to 2010 and a small-scale phase of movement from 2010 to 2018. During the former stage, the northwestern movement of the GDP gravity center was obvious. During the latter stage, the movement range of the GDP gravity center decreased, and its direction of movement shifted to the northeast. During this period, the maximum range of movement exhibited by the GDP gravity center was 13.34 kilometers northwest from 2000 to 2005.

The geographical coordinates of the total retail sales gravity center ranged from 113°39′3″ to 113°46′26″E and from 22°45′24″ to 22°52′44″N, all located in Dongguan. Compared with its location in 2000, the total retail sales gravity center had moved 10.1 kilometers to the northwest by 2018. By the end of the study period, it was closer to the regional geometric center in terms of longitude and latitude. The overall movement of the total retail sales gravity center is similar to that of the GDP gravity center; additionally, it shows a clear trend toward the northwest. During this period, the maximum range of movement exhibited by the total retail sales gravity center was 6.61 kilometers to the northwest from 2005 to 2010.

**Fig 8:**
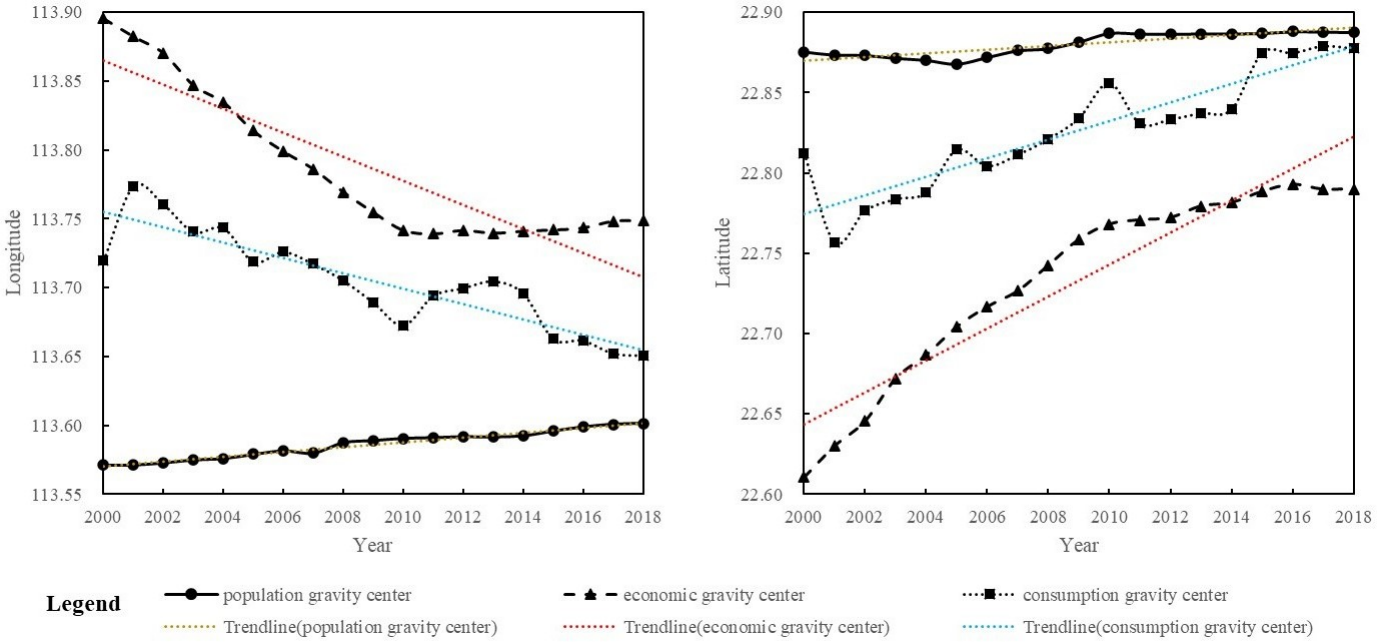
Comparison of the latitudes and longitudes of the population, GDP and total retail sales gravity centers in the GBA.

After decomposing the geographical coordinates of the population gravity center, the longitude and latitude of the GDP and total retail sales gravity centers for each year are calculated as shown in Fig 7 to intuitively explore the changing characteristics and the correlations among the different directions of these three factors’ movements, as shown in Fig 8. Since 2000, the population gravity center of the GBA has generally moved away from the geometric center of the region and has shifted northeast. The GDP gravity center and the total retail sales gravity center show a tendency to approach the geometric center of the region. Compared with their location during the initial stage of the study period, both shifted to the northwest. The latitude range of the population gravity center is close to that of the total retail sales gravity center, and the movement trajectories of the two are convergent.

From 2000 to 2018, the population gravity center in the GBA moved a total of 5.37 kilometers, the GDP gravity center moved a total of 27.41 kilometers, and the total retail sales gravity center moved a total of 39.19 kilometers. In particular, the total retail sales gravity center shows the highest level of movement in the GBA, followed by the GDP and population gravity centers. This indicates that the total retail sales gravity center fluctuates the most, followed by the GDP gravity center; however, the population gravity center is relatively stable.

### The contributions of major cities to the regional differences in the GBA

**Fig 9:**
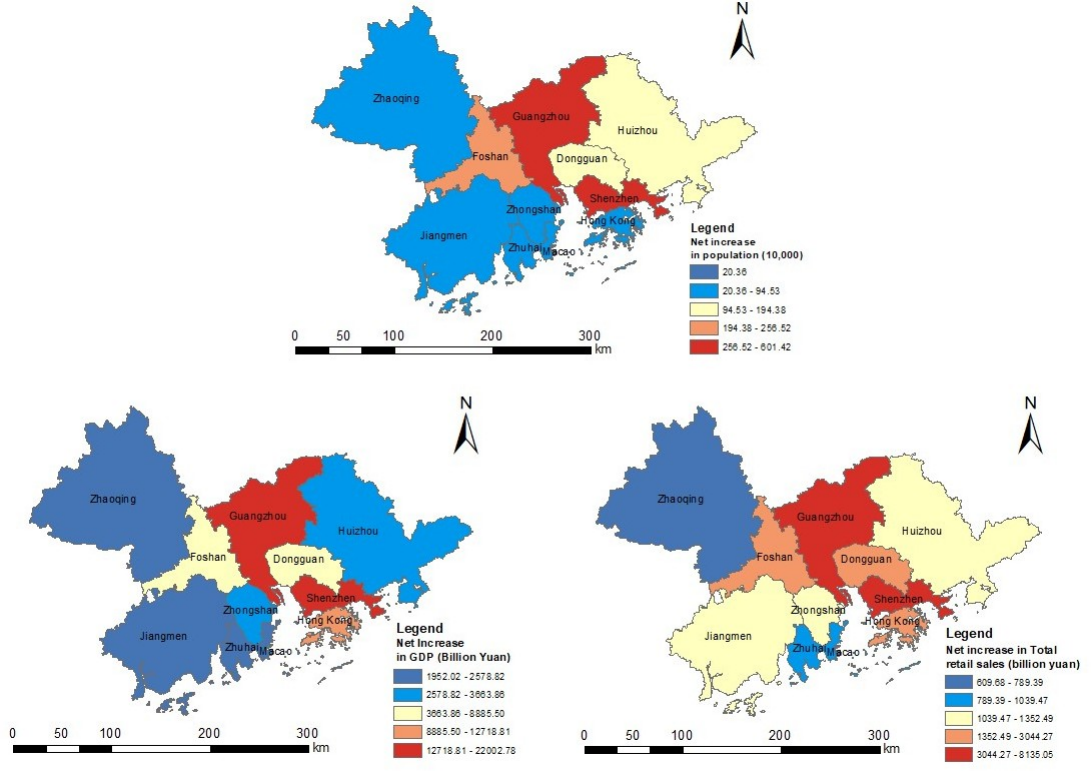
Net increase in the population, GDP and total retail sales of the GBA (2018 vs. 2000). Reprinted background map from the National Catalogue Service for Geographic Information (www.webmap.cn) under a CC BY license, with permission from the National Geomatics Center of China, original copyright 2020.

During the 18 years studied, among the nine cities and the two special zones in the GBA, Shenzhen has experienced the largest population increase, with a total increase of 6.01 million people. As shown in Fig 9, Shenzhen also experienced the highest increase in GDP, with a total increase of 2,200.278 billion yuan. Finally, Guangzhou shows the highest increase in total retail sales over this period, with a total increase of 813.506 billion yuan. Overall, Guangzhou and Shenzhen played leading roles in the regional development of the GBA over the 18 years studied. Due to the differences in the development speeds of the various cities in the region, as well as their different populations and economic carrying capacities, the gaps between the absolute values of the social development indicators of the different cities in the GBA widened from 2000 to 2018.

**Fig 10:**
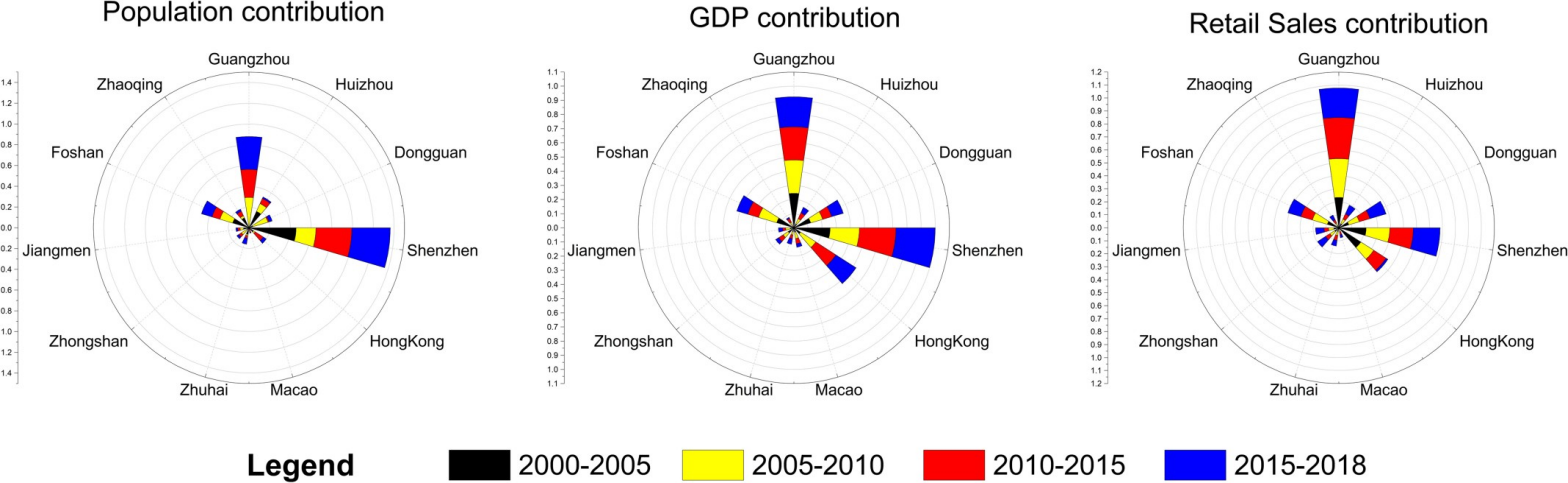
The contribution of each city to the movements of the gravity centers.

To determine which city contributes the most to the movement of the gravity centers, this paper employs statistical data to calculate the contribution of each city to the movement of the different gravity centers during the 2000–2018 period. The results of this analysis are shown in Fig 10. In this rose plot, the length of the color band represents the contribution of the city. Over the 18 years studied, the contribution of the western cities in the GBA to the movement of the population gravity center was less than that of the cities in the central and eastern regions. Among them, Guangzhou and Shenzhen exhibit the highest population increases, followed by Foshan, Dongguan and Huizhou; however, Zhaoqing, Jiangmen, Zhuhai and Zhongshan exhibit the lowest population increases. Since China's reform and opening up, Guangdong has attracted a large number of migrants from the mainland to work in the city and has become a famous manufacturing base. However, due to differences in their administrative systems, economic systems, financial systems, currency systems, etc. [40], as well as their limited land area and high-density population, Hong Kong and Macao do not have much potential to attract large number of mainland Chinese people to live and work there. Therefore, a significant trend in population growth is not observed. In terms of these cities’ contributions to the movement of the GDP and total retail sales gravity centers, three cities of the GBA clearly contribute the most. Guangzhou, Shenzhen and Hong Kong dominate the movement of the GDP and total retail sales gravity centers. Foshan and Dongguan also play important roles, while Zhuhai, Jiangmen, Zhaoqing, Huizhou, and Zhongshan contribute less. Macao’s contribution to the movement of the GDP and total retail sales gravity centers is also small due to its limited economic carrying capacity.

## Discussion

### Discussion of three types of gravity center movement characteristics in the GBA

According to the findings above, the population gravity center gradually moved eastward from 2000 to 2018. As they are first-tier cities of China, Guangzhou and Shenzhen have high-quality social resources and can attract people from all over the country. Dongguan, as it has the advantage of being close to Shenzhen and Hong Kong, has built a globally competitive low-cost industrial zone, thus becoming a "world factory" and attracting a large number of interprovincial floating populations [41]. Huizhou, due to its good environment and low cost of living, has a higher mobility tendency than Shenzhen; thus, it has accommodated the housing needs of some Shenzhen workers. In contrast, the western region lacks leading cities; Foshan’s population contribution is not as advantageous as that of the cities in the eastern region, and it does not have enough power to pull the center of population toward the west. It should be noted that the population, GDP and total retail sales gravity centers are related to the regional average centers instead of the absolute higher values.

With regard to the changing trends exhibited by GDP and total retail sales, the GDP and total retail sales gravity centers show similar spatiotemporal trends; both moved significantly northwestward during the 2000–2018 period [42, 43]. Specifically, the GDP gravity center was far from the regional geometric center in 2000 and was very close to Hong Kong. In fact, Hong Kong’s GDP was in an absolute leading position in the GBA in 2000; its GDP was 4.5 times that of Guangzhou and 5.1 times that of Shenzhen. However, from 2000 to 2018, Hong Kong’s GDP increased at a much slower rate. Shenzhen overtook Hong Kong in terms of GDP for the first time in 2018, and the gap between Guangzhou’s GDP and Hong Kong’s GDP was only 114.163 billion yuan. Moreover, after the period of prosperity experienced by Hong Kong, its status as an economic center declined; subsequently, serious industrial hollowing problems occurred [44]. The growth rate of Hong Kong’s GDP was not as good as that of Guangzhou and Shenzhen.

### Temporal comparison of GBA evolution

The GBA is one of the most dynamic economic regions and manufacturing centers in the world [45]. However, before 1978, except Guangzhou, the other cities in Guangdong had agriculture as their pillar industry [46], and Shenzhen in particular was a small village that relied on fisheries [47]. At this time, Hong Kong and Macao were under the rule of Britain and Portugal, respectively. They relied on light manufacturing to lead the development of the GBA [48]. There is no doubt that the development gravity center of the GBA at this time is clearly leaning toward Hong Kong and Macao.

After 1978, the central government of China adopted a series of policy reforms. The cities in Guangdong Province developed an export-oriented economy with cheap labor and land resources and undertook an extensive transfer from various industries, including manufacturing in Hong Kong and Macao. At this time, Hong Kong is still the core of the GBA [49]. It is conceivable that at this time, the development gravity center of the GBA is still biased toward Hong Kong, but with the influence of the Mainland's reform and opening-up policy, the regional development gravity center will inevitably move northward.

With China's efforts to expand its opening up and economic reform after joining the WTO at the end of 2001, the cities of Guangdong Province have ushered in a new momentum of development. Dongguan, which is known as "an important information technology R&D center", and Foshan, which aims to become "an advanced manufacturing base", have especially attracted and developed advanced manufacturing industries and achieved tremendous development [50]. During this period, Guangzhou and Shenzhen were also developing rapidly, so it is obvious that the gravity center of regional development shifted northward.

### Policy recommendations regarding the development of GBA

To achieve balanced regional socioeconomic development, the individual cities in the GBA megacity region need effective policies. Based on the findings above, we propose several policy suggestions for the individual cities, as follows: (1) while maintaining high-quality development, Guangzhou and Shenzhen should further connect with other cities to promote their common development; they should move from "struggling singly" to "cooperative operations" and help underdeveloped regions address their shortcomings; (2) Hong Kong and Macao should gradually break down the institutional barriers between themselves as special administrative regions and the mainland, making full use of the mainland’s market, capital, talent and technology and strengthening the "one country, two systems" exchange in the context of the GBA; (3) Foshan and Dongguan should maintain their current positive development momentum, further enhance the development potential of their manufacturing industries, and continue to support the development of high-tech industries; (4) by implementing policies, the GBA should encourage the development potential of cities such as Zhaoqing, Jiangmen, Zhongshan, Huizhou and Zhuhai, enhance the construction of infrastructure, accept industrial transfer to developed areas, promote industrial transformation and upgrades, offer more job opportunities, and stabilize the local population’s employment and living conditions in the local area. Additionally, the cities of the GBA should work to increase their floating populations, promote mutual increases in population, economic factors and consumption, and promote the balanced development of the GBA.

## Conclusion

Overall, the differences in geography, transportation, policies and the other factors present within the GBA have led to differences and constant changes in its socioeconomic development, as well as an imbalance in its regional development. The sustainable development of the region cannot rely solely on the guidance of a few cities. In that case, the lagging development of other cities would definitely put a considerable amount of pressure on the development of the entire region. The goal of this paper is to investigate the imbalances in the regional development of the GBA over a long period of time. Thus, this paper analyzes the spatiotemporal evolution of the regional socioeconomic development of the individual cities in the GBA region from 2000 to 2018. The main conclusions of this paper can be summarized as follows.

Based on their different foundations and development characteristics, the cities in the GBA can be divided into four groups, as follows: Hong Kong (high starting point and low growth rate); Guangzhou and Shenzhen (high starting point and high growth rate); Foshan and Dongguan (low starting point and high growth rate); Macao, Zhuhai, Zhongshan, Zhaoqing, Huizhou, and Jiangmen (low starting point and low growth rate).

From 2000 to 2018, the population densities of the central and eastern regions of the GBA were higher than that of the western region; additionally, the GDP and total retail sales of the central and eastern regions were higher than those of the western region. GDP shows the greatest imbalance in terms of distribution in the GBA, and it is followed by total retail sales and population; additionally, total retail sales exhibits the highest level of movement and is followed by GDP and population.

From 2000 to 2018, Guangzhou, Shenzhen and Hong Kong contributed the most to the changes in the socioeconomic gravity centers of the GBA, while Foshan and Dongguan both played important roles.

Based on the findings above, future studies will focus on the GBA and explore the regional distribution, circulation, transformation, and gravity center changes of different industries in the GBA over a long period of time (including labor-intensive industries, resource-intensive industries, capital-intensive industries, knowledge-intensive industries, etc.). Moreover, land use data will be introduced to analyze the relationship between industrial changes and land use changes.
